# Development and validation of a HPLC-UV method for methadone hydrochloride quantification in a new oral solution with preservatives to be implemented in physicochemical stability studies

**DOI:** 10.1186/s13065-022-00827-9

**Published:** 2022-05-14

**Authors:** Elena Alba Álvaro-Alonso, Mª Paz Lorenzo, Ismael Escobar-Rodríguez, Antonio Aguilar-Ros

**Affiliations:** 1 Pharmacy Department, Infanta Leonor University Hospital, Av. Gran Vía del Este, 80, 28031 Madrid, Spain; 2 Center for Metabolomics and Bioanalysis (CEMBIO), Facultad de Farmacia, Universidad CEU San Pablo, CEU Universities, Urbanización Montepríncipe, 28660 Boadilla del Monte, Spain; 3 Facultad de Farmacia, Universidad CEU San Pablo, CEU Universities, Urbanización Montepríncipe, Boadilla del Monte, 28660, Spain

**Keywords:** Methadone hydrochloride, Pharmaceutical solutions, Drug compounding, High performance liquid chromatography, Analysis, Analytical chemistry method

## Abstract

**Purpose:**

The Pharmacy Service of the Infanta Leonor University Hospital acquires, compounds, distributes and dispenses more than 3000 L of methadone oral solution to Drug Addiction Patients Centers per year. Our purpose is to develop and validate an improved high performance liquid chromatography (HPLC) method to quantify methadone hydrochloride in a new oral solution with methylhydroxybenzoate (methylparaben) and propylhydroxybenzoate (propylparaben) to be implemented in physicochemical stability studies that allow to provide more information and even to increase the beyond-use date.

**Methods:**

A HPLC-Agilent^®^ 1100 equipment, comprising a quaternary pump and an ultraviolet diode-array-detector (DAD) was used. An analytical method development and validation was completed. The curve was constructed from methadone working concentrations of 75–125% (7.5, 9.0, 10.0, 11.0 and 12.5 mg/mL) to assess the linear relationship between the concentration of the analyte and the obtained areas. Precision and accuracy were calculated. Detection and quantification limit (LD, LQ) were estimated using the EURACHEM method. Forced-degradation studies were also performed.

**Results:**

Chromatographic conditions were: flow rate 1.6 mL/min; mobile phase 55% acetonitrile and 45% sodium phosphate 25 mM (pH = 10); injection volume was 5 µL. The column was a Waters-XTerra™ RP18, maintained at 40 °C. DAD was λ = 254 nm. Retention times for methadone, methylparaben and propylparaben were 4.34, 0.70 and 0.88 min respectively.

The method was linear (y = 284.3x − 97.8, r = 0.996). Instrumental precision was 0.33% for standards (n = 10); intra-assay precision 0.53% (n = 6) and inter-assay precision 1.95% (n = 12). The relative standard deviation percentage for accuracy was 1.28%. The recovery percentage was 101.5 ± 1.5%. LQ and LD were 2.18 µg/mL and 2.0 µg/mL respectively. The most destabilizing conditions were oxidizing and alkaline. The chromatograms confirmed no interference with the methadone signal.

**Conclusions:**

The HPLC method has proved to be valid and reproducible for methadone quantification in a new oral solution with methylparaben and propylparaben. This assay is a rapid, simple and reliable technique that can be used in daily analysis and physicochemical stability studies.

## Introduction

In the Autonomous Community of Madrid, Resolution 189/2018 [1] was implemented in March 2018, which tasked the Hospital Pharmacy Service (HPS) of the Infanta Leonor University Hospital with supplying methadone to the 27 Centres for the Comprehensive Care of Drug Addiction Patients of the Madrid Health Care Service where methadone maintenance programs (MMP) for opiate addictions are implemented. The aim of this Resolution was to centralize the acquisition, preparation, distribution, and dispensing of methadone by the HPS. This initiative represented a first step in changing the pharmacotherapeutic health care model for the treatment of the patients in the program. To date, between 3000 and 5000 patients are prescribed methadone as an opiate substitute for the treatment of heroin-related addiction disorders.

The methadone solution prepared and supplied by the HPS to MMP patients is described in the Spanish National Formulary [2] and is formulated with methadone hydrochloride in the raw material form and purified water. A beyond-use date (BUD) of 30 days and refrigerated storage have been established. For this reason, and due to the large volume of methadone solution to be dispensed (around 3500 L per year), one of the future challenges [3] consists in carrying out a physicochemical and microbiological stability study in order to confirm and even increase its BUD as well as the development of improvements to the formulation of the methadone solution by adding preservatives. Thus, a new compounding of methadone hydrocloride in oral solution was designed and validated. Its composition included methylhyparaben and propylparaben as preservatives. The final concentration was also 10 mg/mL.

It was necessary to determine and quantify the methadone hydrochloride in the new oral solution without interference from the preservatives.

Methadone is an extensively studied drug and according to the Spanish Pharmacopoeia [4], the technique of choice is Gas Chromatography (GC). However, in the United States Pharmacopoeia [5] (USP), quantitation methods for methadone in pharmaceutical preparations are based on acid titration, UV determination, GC, and High Performance Liquid Chromatography (HPLC). Some of these methods present, as do HPLC methods described in the literature for methadone analysis, the following disadvantages: lenghthy and wasteful use of solvent analysis (gradient elution over 10–20 min), a tedious extraction procedure (not suited for routine analysis) or the use of a fluorescent ion-pairing agent [6–10].

It should be note that the methadone analyzed with these methods was either found in concentrations different from 10 mg/ml, or they were preparations for intravenous administration, or they were compounded with other vehicles than water (sodium chloride [11, 12], various drinks [13], syrups, suspension and sugar-free vehicles [14]) and none of the pharmaceuticals analyzed for oral administration contained exactly the same preservatives (only methylparaben) [9] used in our new formulation [15], so they really were not suitable for us. Furthermore, these methods use columns that are no longer used, such as µBondapak or Radpak-Novapak, a type A silica-based column with a lot of silanolic activity and therefore a high possibility of deformation tailing.

Other studies performed since 2000 were focused on the development of new techniques to determine methadone in biological samples such as plasma, saliva and urine [16, 17] and even in wastewater [18–20], or by HPLC-Ion-Trap Mass Spectrometry [21], electrochemical detection [22] or other extraction techniques. These methods, despite being more precise and having been developed in more complex matrices than our solution, are more complex and expensive techniques. Therefore, there would not be suitable for our work. Methadone stability studies have also been described, but the methadone determination technique is either the USP one, those previously shown in the literature, GC [23] or spectrophotometry [14].

New analytical methods are always sought in order to obtain more and better information, with less consumption or contamination, in less time and with less effort. In addition, analytical method development and validation procedures are vital in the discovery and development of drugs and pharmaceuticals to ensure the method performance.

The aim of this study was to develop and validate a simple, rapid and reproducible analytical method to quantify methadone hydrochloride in a new oral solution with methylparaben and propylparaben to be implemented in physicochemical stability studies. The future goal is to increase the BUD of methadone hydrochloride oral solution to improve organizational aspects of the workflow of HPS and increase adaptability to the individual dispensing needs of patients. It will also be necessary to carry out a physicochemical and microbiological stability study in different conservation environments of the new formulation proposed in this work to provide more information.

## Methods

### Reagents, reference standards and materials

It has been used methadone hydrochloride purchased from Laboratorios Dr. Esteve S.A. (Barcelona, Spain). As preservatives, methylparaben and propylparaben, acquired from Fagron Iberica (Terrassa, Spain) were used. Purified water was obtained from Grifols laboratory (Barcelona, Spain). All of them were of Pharmacopoeia grade.

In the mobile phase we used acetonitrile HPLC grade, purchased from VWR Prolabo Chemicals (Fontenay-Sous-Bois, France). Phosphoric acid, sodium hydroxide (> 99%), hydrochloric acid and hydrogen peroxide were supplied from Panreac (Barcelona, Spain) and Milli-Q water. All reagents and solvents were of analytical grade.

### Equipment

HPLC analyses were performed on a qualified and calibrated chromatography system, Agilent-Technologies 1100 series (Madrid, Spain) comprising a quaternary gradient pump, an ultraviolet photodiode-array detector (UV-DAD), a 100-vial programmable autosampler, a column oven compartment, an automatic injector and a software controller.

### Chromatographic conditions

We have used a Waters-XTerra^TM ^RP18 (3.5 μm;4.6 × 100 mm) column. The column temperature was maintained at 40 °C. The mobile phase consisted of acetonitrile as the organic phase (55%) and sodium phosphate 25 mM (adjusted to pH = 10) as the aqueous phase (45%). The flow rate was 1.6 mL/min. The injection volume was 5 µL for each chromatographic analysis. The UV-DAD was set at λ = 254 nm.

### Validation of the HPLC method

The methods and their acceptance criteria were established on the basis of the International Conference on Harmonization (ICH) guidelines Q2 (R1) [25].

#### Linearity

A standard solution (100 mL) of methadone 50 mg/mL was prepared from which, by means of serial dilutions, a total of 20 calibration standards were obtained for the linearity test (four replicates for each concentration level). The curve was constructed from methadone working concentrations of 75–125% (7.5, 9.0, 10.0, 11.0 and 12.5 mg/mL) to assess the linear relationship between the concentration of the analyte and the obtained areas. Once the regression equation was obtained [26], an analysis of variance (ANOVA) was performed.

#### Precision

Instrumental precision (repeatibility), intra-assay precision and inter-assay precision (intermediate precision) were measured. For instrumental precision, a standard solution (10 mL) of methadone 10 mg/mL was prepared by the same analyst on a single day and consecutively analyzed ten times to check the repeatability of the method and to assess the dispersion degree among the series of measurements obtained. For intra-assay precision, six standards of methadone solution 10 mg/mL were prepared and analyzed. Inter-assay precision was also performed in another six standards of 10 mL of methadone solution 10 mg/mL which were prepared on a second day by different analysts, obtaining a total of 12 samples.

#### Accuracy

The accuracy of the method was determined through spike recovery of the methadone solution with a preservative matrix, diluted within the range used for final sample measurements, and within the range of the corresponding calibration curves. Afterwards, three 10 mL replicates of three concentration levels 7.5, 10 and 12.5 mg/mL were prepared by serial dilutions from 100 mL of a 50 mg/ml stock solution. The recovery percentage and relative standard deviation percentage (%RSD) were calculated. The maximum aceptable levels were 10%.

### Detection and quantification limit

Detection limit (LD) and quantification limit (LQ) in case of instrumental method, can be estimated using various equations. However, in this assay, it was decided to use an experimental method, following EURACHEM recommendations [27] which consisted of preparing a series of samples with decreasing amounts of analyte and analyzing each of them six consecutive times, representing %RSD of the precision against the concentration of each sample. For this purpose, we prepared a battery of serial dilutions from a stock solution of methadone 20 mg/mL. The concentrations were 2, 0.2, 0.02, 0.002 and 0.0002 mg/mL. Six replicates of each concentration were prepared, from which the area and retention times were obtained. Normally, a precision criterion of %RSD of 10% is set at the LQ although up to 20% can be accepted, depending on the characteristics of the method. Both were also expressed as a percentage of the theoretical concentration.

### Forced-degradation studies

The methadone hydrochloride 10 mg/ml oral solution was subjected to the following denaturing conditions to determine the capacity of the HPLC method in order to detect any possible degradation products produced during storage: in acid (0.1 M HCl at 25 °C), in base (0.1 M NaOH at 25 °C) and in oxidation (3% H_2_O_2_ at 25 °C). For this, 0.1 mL of methadone 10 mg/mL was diluted in 1 mL of each denaturing reagent and they were kept in contact for 1 h until analysis. Then, following the same chromatographic conditions, elution cycles of 60 min were programmed, and the test was carried out eight times for each stress condition. Peak purity was also calculated using the Agilent-ChemStation software tool based on the similarity factor.

## Results

**Fig. 1:**
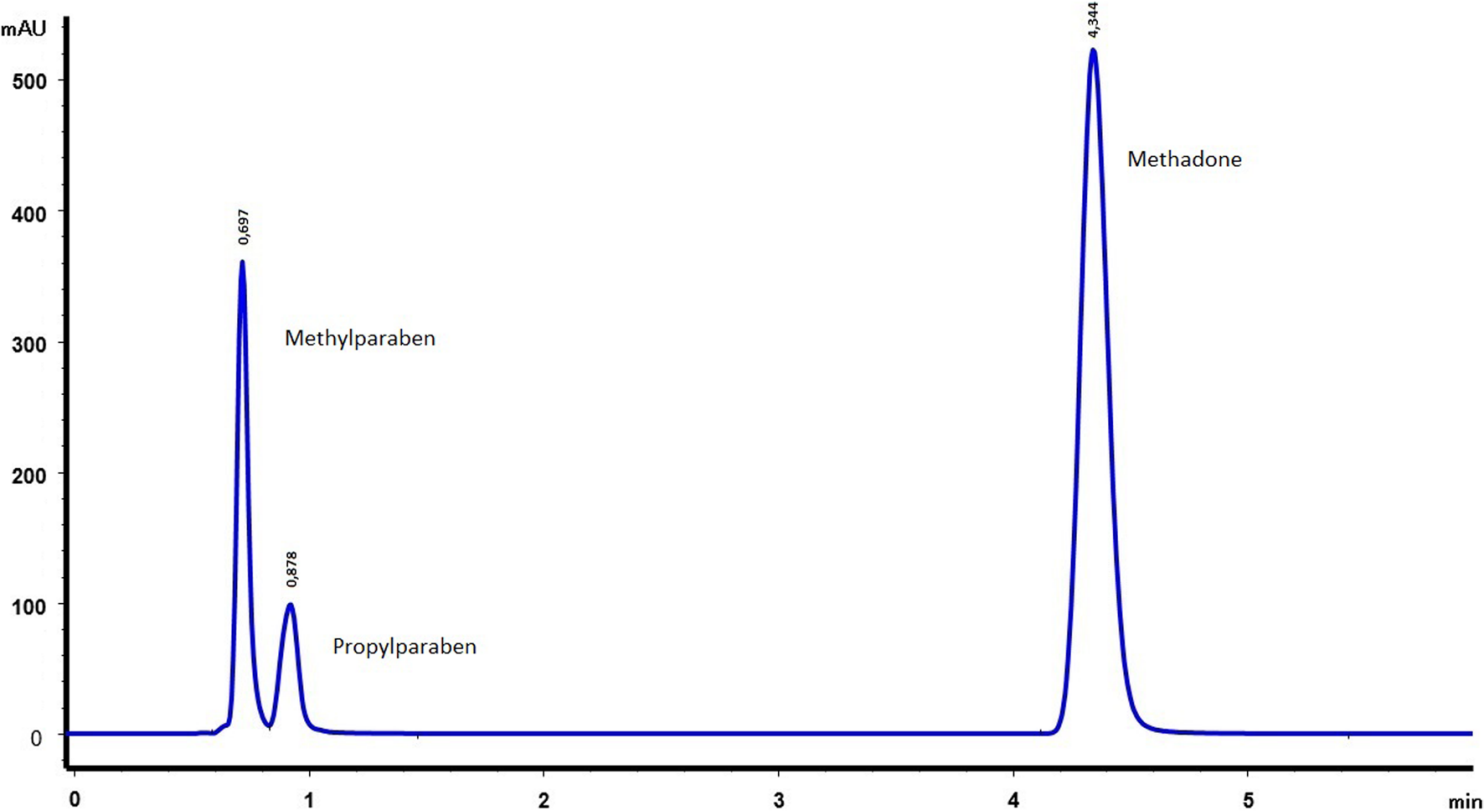
Chromatogram of methadone hydrochloride 10 mg/mL with preservatives

**Fig. 2:**
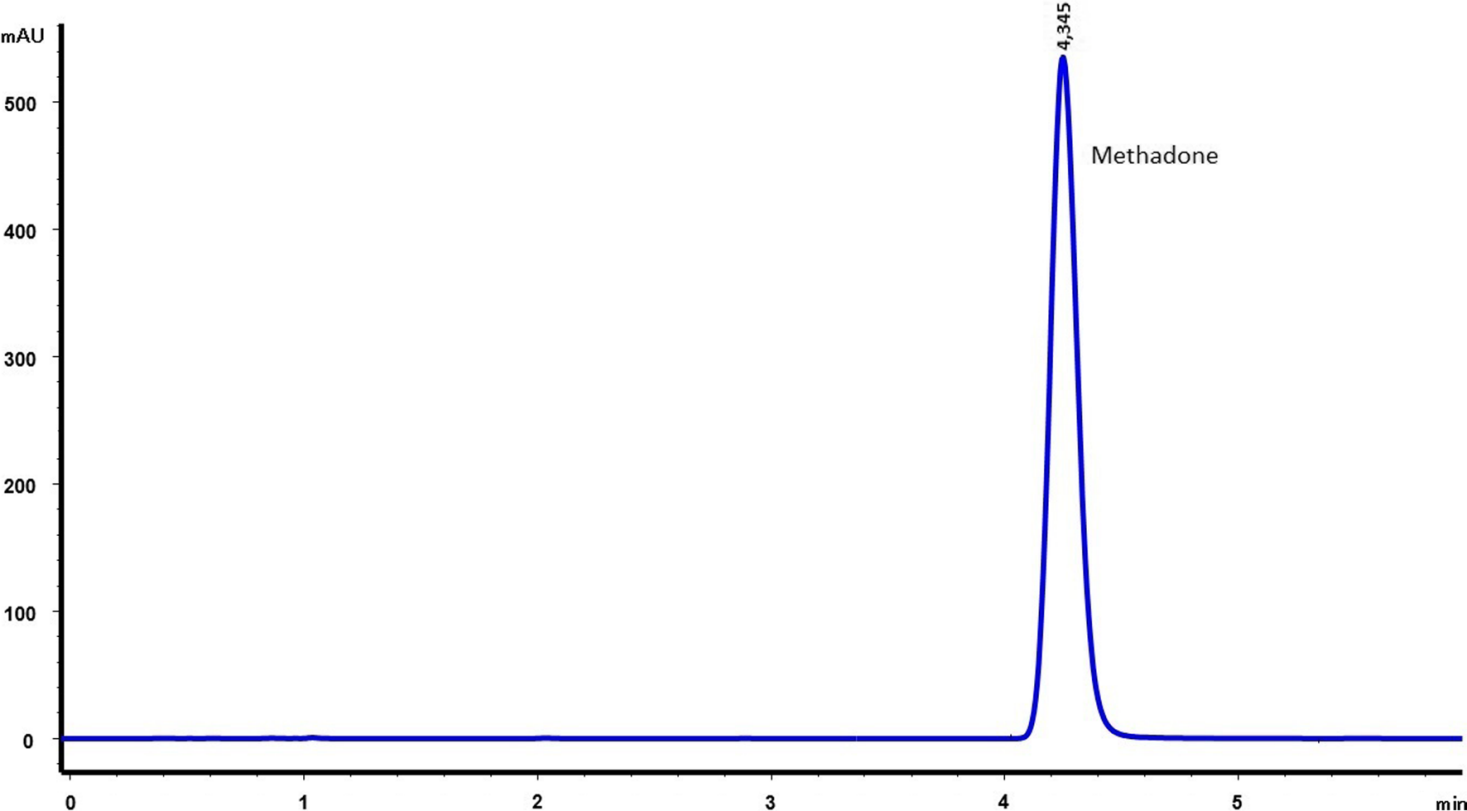
Chromatogram of methadone hydrochloride 10 mg/mL without preservatives

**Fig. 3:**
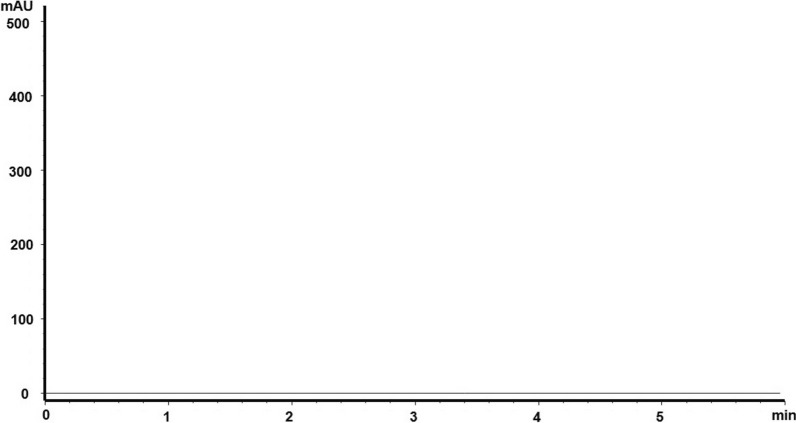
Chromatogram of blank

The HPLC method has demonstrated that there is no substance that interferes in the analysis of the different formulations of methadone. It has been verified that, at the analysis wavelength, 254 nm, there is no interference with the parabens. In our analysis conditions, methadone appears at a retention time of 4.34 min (which is 50% less than in the USP monograph) [5] and methylparaben and propylparaben at 0.70 and 0.88 min respectively. The chromatograms obtained are shown in Figs. 1, 2 and 3.

### Linearity

ANOVA analysis confirmed the linearity of the method in the range tested. The equation obtained was y = 284.3x − 97.8 being the correlation coefficient (r) 0.996 and determination coefficient (r^2^) of 0.991. The results indicate that there is no significant statistical dispersion between the results of the replicates of the different concentrations, with a correlation and determination coefficient greater than 0.99, corroborating compliance with the linearity method.

### Precision and accuracy

The results of instrumental precision, intra-assay and inter-assay precision and accuracy are shown in Tables 1, 2. The percentage of recovery for all samples fulfilled the requirements of the compounding stability studies (90–110%) [28]. The small percentage of difference between the nominal and found concentration of the standards showed that the assay is sufficently accurate for their application. In addition, the %RSD value was below 10% at all concentrations, indicating that the assay method was reproducible across days.

**Table 1:** Accuracy results

	Sample	Concentration (mg/mL)	Area	Recovery percentaje
Intra-assay accuracy	1	7.5	2156.23	101.77%
	2		2156.13	101.76%
	3		2150.20	101.50%
	4	10	2834.91	99.30%
	5		2792.10	97.85%
	6		2838.16	99.41%
	7		2843.81	99.60%
	8		2852.00	99.88%
	9		2864.73	100.31%
	10	12.5	3526.82	98.18%
	11		3599.04	100.14%
	12		3606.48	100.34%
			*Median*	*100.00%*
			*%RSD*	*1.27%*
Second-assay accuracy	1	7.5	2044.37	96.72%
	2		2034.18	96.26%
	3		2015.79	95.43%
	4	10	2725.24	95.59%
	5		2719.33	95.39%
	6		2714.90	95.24%
	7		2718.15	95.35%
	8		2713.72	95.20%
	9		2733.81	95.88%
	10	12.5	3520.51	98.01%
	11		3527.89	98.21%
	12		3532.32	98.33%
			*Median*	*96.30%*
			*%RSD*	*1.22%*
	Inter-assay accuracy		Median	98.15%
			%RSD	1.24%

**Table 2:** Precision and accuracy results

Instrumental precision (%CV)	Intra-assay precision			Inter-assay precision		
	Precision (%CV)	Accuracy (%)	RSD (%)	Precision (%CV)	Accuracy (%)	RSD (%)
0.33 (n=10)	0.5 (n=6)	100	1.3	2.0 (n=12)	98.15	1.2

### Detection and Quantification Limit

After representing %RSD versus concentration, a potential adjustment was observed. The equation obtained was y = 0.0051x^−0.37^. We observed that as the concentration decreases, %RSD increases due to the difficulty of detecting methadone. For 0.002 mg/mL, %RSD was 11.3%. For 0.0002 mg/mL, there were four samples with no signal, so we were unable to obtain the area value.

Interpolating in the obtained curve a %RSD = 10%, we calculated that LQ was 2.18 µg/mL. The LD is the value capable of detecting the analyte. However, in the 0.0002 mg/mL concentration, four of the six samples were not detected, so the LD was considered to be the previous concentration value in which methadone was detected. Therefore, LD was 2.0 µg/mL. Expressing these values as a percentage of the theoretical concentration, LQ and LD were 0.022% and 0.02%. This means that the method is capable of detecting up to 0.02% and quantifying 0.022% of methadone contained in a 10 mg/mL oral solution (the objective of our method was to quantify methadone at around 100% concentration).

### Forced-degradation studies

The results show that the method was stability-indicating, with complete separation of the degradation products from our drug peak of interest (in all situations, the methadone peak continued to be obtained at minute four). The methadone peak purity was 999,830 over 1000 (1000 indicates identical spectra and values > 995 indicate that the spectra are very similar).

**Fig. 4:**
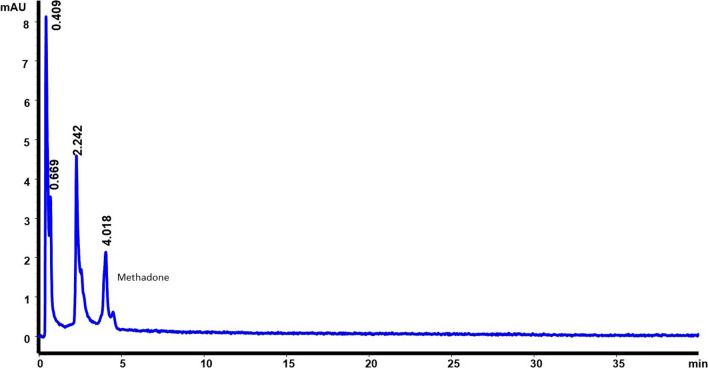
Chromatogram in base condition

**Fig. 5:**
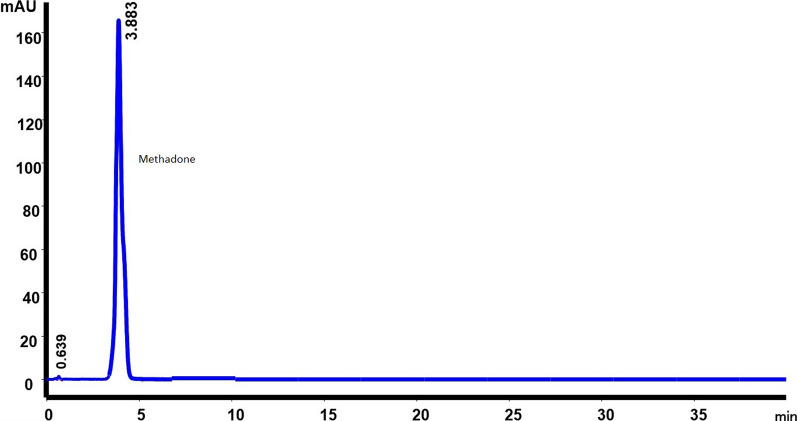
Chromatogram in acid condition

**Fig. 6:**
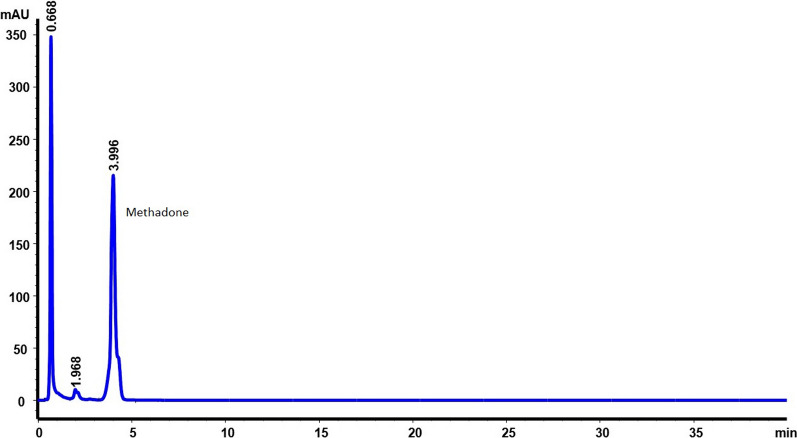
Chromatogram in oxidizing condition

The most destabilizing conditions were oxidizing and alkaline. In basic medium, turbidity was observed practically instantaneously, indicating insolubility of the methadone under this condition. In an acidic and oxidative environment, some degradation of methadone was observed due to the fact that we obtained recovery percentages of 88%. Figures 4, 5, 6 show the chromatograms obtained which confirmed that none of the peaks found interfered with the methadone signal and that no degradation products appear after 60 min. This means that the method is capable of quantifying methadone separated from degradation products.

## Discussion

A full method validation should be performed for any analytical method whether new or based upon literature [29] as this ensures that the method developed is reproducible, stable, sensitive, robust, suitable and reliable for its application in pharmaceutical analysis.

The ICH recommends evaluating linearity in the range 80–120% [25]. In our case, a margin of 75–125% was chosen and the method demonstrated good linearity over the range assayed. The method was repeatable. Intra- and inter-assay precision %RSD were < 10% indicating that the assay method was reproducible across days. The accuracy of an analytical procedure expresses the closeness of agreement between the value which is accepted either as a conventional true value or an accepted reference value and the value found [25]. The small percentage of difference between the nominal and found concentration of the standards demonstrated that the assay is accurate enough for its application. The mean recovery values obtained were 100.0% and 98.5% respectively. Higher recovery indicates an efficient extraction procedure and higher sensitivity and accuracy of the analytical method.

Following the specifications for methadone hydrochloride in oral solution described in the USP, Spanish Pharmacopoeia and Spanish National Formulary [2, 4, 5], it contains no less than 90.0% and no more than 110.0% of methadone hydrochloride. Consequently, one of the objectives of our method was to quantify methadone near 100% concentration.

When the method is defined as a content assessment analysis, where we will always work in ranges distant from the minimum detectable or quantifiable quantity using the equipment, it is not necessary to determine LQ and LD. However, they were calculated, on the one hand to demonstrate this situation, and on the other hand, to have a more complete validation because it allows a better understanding of the analytical method, and knowing the minimum analyte amounts that can be quantified, can be useful for other applications. The method has been shown to be capable of detecting and quantifying at the value of 0.02%, so the method not only quantifies perfectly in the limit of 90–110% indicated by the pharmacopoeias, but also is capable of quantifying impurities or degradation products that could be present in 0.02% of the final sample analyzed, in our case, the new oral solution.

The ability of the assay to detect methadone decomposition was demostrated by stressing a methadone sample in forced-degradation studies which showed that the most destabilizing stress conditions were the oxidizing and alkaline conditions. The peaks obtained did not interfere with methadone. Therefore, we can infer that under pH conditions according to the specifications (< 6.5), the pH variation within said limits will not produce any product that interferes with methadone quantification. Thus, it has been demonstrated that our method is suitable for the detection and quantification of methadone hydrochloride in the presence of degradation products. It is important to highlight that methadone solution as final pharmaceutical product will never occur in these extreme conditions, so it is unncessary to characterize and quantify the peaks found in the chromatograms.

On the other hand, to understand the chromatographic conditions chosen for this study, it is first necessary to study the characteristics of the methods described in the literature, and secondly, to know the methadone hydrochloride behavior in order to improve the conditions described. In this sense, at the beginning of the method design, we studied methadone characteristics, and based on this, we tested different conditions to choose the more suitable ones.

The methadone pKa is 8.3, so a high pH environment was needed in which we make sure that methadone is totally deprotonated. It was necessary to choose a suitable column and a mobile phase to work at a high pH values. For this reason, the column chosen was the Waters-XTerra^TM ^RP18, which in addition to being resistant (it allows working at pH up to 12), allowed us to avoid the tailing factor, obtain short retention times and achieve a good resolution of the methadone peak, in addition to being sufficiently effective in avoiding possible interference between methylparaben, propylparaben and methadone (Fig. 1). The XTerraRP (First-Generation-Hybrid-Filler) columns, combine the best properties of silica and polymer bonded phases with patented hybrid-particle-technology, which replaces one in three silanols with a methyl group. The result is a mechanically strong particle that can be used for high pH separations; as a consequence the charge and peak shapes of the basic compounds are improved.

Our wavelength is the one collected inside the UV-DAD and in USP method, where comparing with 274 nm, a higher absorption was observed. Regarding the mobile phase, different percentages were tested until 45–55% was reached, where the peaks were perfectly separated and resolved in < 5 min.

The evaluation of robustness was considered not necessary. Variations in the flow rate or temperature will not put the resolution between paraben and methadone at risk, as the resolution between them is close to 20. Nevertheless, the pH of the mobile phase is a critical parameter due to the ionization of methadone and the stability of the stationary phase. According to the pKa of methadone and the column manufacturer´s directions, pH must be adjusted between 9.3 and 11. Below 9.3 methadone peak will show lower retention time and tailing, and above 11 the shelf life of the column is diminished.

In this study we needed an analytical method to quantify methadone hydrochloride in a new oral solution with preservatives, not previously analyzed in the literature. Comparing our results with those found in other HPLC methods developed in different methadone formulations, we can affirm that our method is efficient, rapid, simple, capable of quantifying methadone without interference from preservatives and better than the methods already described whose disadvantages have been highlighted.

The method reported in this assay can also be used to carry out physicochemical stability studies in which the possible methadone degradation can be detected in different conservation environments over a period of time.

## Conclusions

The HPLC method reported in this study is a rapid, simple, reliable and economical technique analytically validated and has allowed for the efficient quantification of methadone hydrochloride in a new oral solution with methylparaben and propylparaben as preservatives and a concentration and composition not previously analyzed in the literature. This procedure is a new and an improved method in comparison to those described in the USP and literature. It has great recovery and the advantage that the ion-pairing technique was not required, thus saving time and money, which are key aspects in these times.

Furthemore, the HPLC method reported can be used in the daily analysis of the methadone solution batches prepared in the HPS and also to perform physicochemical stability studies in different conservation environments over a period of time in order to increase the BUD.

## Data Availability

The datasets used and/or analysed during the current study are available from the corresponding author on reasonable request.

## References

[1] Resolución del director general de coordinación de la asistencia sanitaria por la que se encomienda al Hospital Universitario Infanta Leonor las tareas de suministro diario de metadona a los centros de atención integral a drogodependientes de la Subdirección General de Asistencia en Adicciones del Servicio Madrileño de Salud. Dirección General de Coordinación de la Asistencia Sanitaria, n^o^ 189/2018, (27 de febrero de 2018).

[2] Formulario Nacional. 3^a^ Ed. 2020.https://www.aemps.gob.es/laAEMPS/docs/formulario-nacional.pdf?x59481&x60265

[3] Álvaro-Alonso EA, Tejedor-Prado P, Aguilar-Ros A (2020). Centralization of the methadone maintenance plan in a hospital pharmacy department in the Community of Madrid. Farm Hosp.

[4] Metadona, hidrocloruro de (01/2008, 0408). Real Farmacopea Española en internet. 5^a^ ed. 2015.https://extranet.boe.es/farmacopea/doc.php?id=0408

[5] The United States Pharmacopeia: USP–NF 2021*, *2021; (3).

[6] Beasley TH, Ziegler HW (1977). High-performance liquid chromatographic analysis of methadone hydrochloride oral solution. J Pharm Sci.

[7] Hsieh J, Ma J, O’donell J (1978). High-performance liquid-chromatographic analysis of methadone in sustained-release formulations. J Chromatogr.

[8] Derendorf H, Garrett ER (1983). High-performance liquid chromatographic assay of methadone, phencyclidine, and metabolites by postcolumn ion-pair extraction and on-line fluorescent detection of the counterion with applications. J Pharm Sci.

[9] Adams P, Haines-nutt R (1985). High-performance liquid-chromatographic analysis of methadone hydrochloride in pharmaceuticals. J Chromatogr.

[10] Helmlin HJ, Bqurquin D, De Bernardini M (1989). Determination of methadone in pharmaceutical preparations using high-performance liquid chromatography with photodiode array detection. Pharm Acta Helv.

[11] Soy D, Roca M, Deulofeu R (1998). Stability of 0.1% and 0.5% oral methadone clorhydrate solutions in saline. Farm Hosp.

[12] Friciu MM, Alarie H, Beauchemin M (2020). Stability of methadone hydrochloride for injection in saline solution. Can J Hosp Pharm.

[13] Lauriault G, Lebelle M, Lodge B (1991). Stability of methadone in 4 vehicles for oral-administration. Am J Hosp Pharm.

[14] Provenza N, Calpena AC, Mallandrich M (2016). Design of pediatric oral formulations with a low proportion of methadone or phenobarbital for the treatment of neonatal abstinence syndrome. Pharm Dev Technol.

[15] Ching MS, Stead ChK, Shilson AD (1989). Stability of methadone mixture with methyl hydroxybenzoate as a preservative. Aust J Hosp Pharm.

[16] Miguez-Diez E, Pitarch-Sierra A, Modamio P (2013). HPLC-UV method development and validation for quantitative determination of methadone in human plasma. Int J Clin Phar.

[17] George R, Lobb M, Haywood A (2016). Quantitative determination of the enantiomers of methadone in human plasma and saliva by chiral column chromatography coupled with mass spectrometric detection. Talanta.

[18] Castiglioni S, Zuccato E, Crisci E (2006). Identification and measurement of illicit drugs and their metabolites in urban wastewater by liquid chromatography−tandem mass spectrometry. Anal Chem.

[19] Baker DR, Kasprzyk-Hordern B (2011). Multi-residue determination of the sorption of illicit drugs and pharmaceuticals to wastewater suspended particulate matter using pressurised liquid extraction, solid phase extraction and liquid chromatography coupled with tandem mass spectrometry. J Chromatogr A.

[20] Foppe KS, Subedi B. Analysis of Illicit Drugs in Wastewater Using High-Performance Liquid Chromatography-Electrospray Ionization-Tandem Mass Spectrometry (HPLC-ESI-MS/MS). In: Musah RA, ed. Analysis of Drugs of Abuse. New York: Springer New York 2018. 183–91. Doi:10.1007/978-1-4939-8579-1_16 10.1007/978-1-4939-8579-1_16 29974428

[21] Musile G, Cenci L, Piletska E (2018). Development of an in-house mixed-mode solid-phase extraction for the determination of 16 basic drugs in urine by High Performance Liquid Chromatography-Ion Trap Mass Spectrometry. J Chromatogr A.

[22] Kokubun H, Takigawa C, Miyano K (2018). A novel method for determination of methadone in the serum by high-performance liquid chromatography with electrochemical detection. Biol Pharm Bull.

[23] Denson DD, Crews JC, Grummich KW (1991). Stability of methadone hydrochloride in 0.9% sodium chloride injection in single-dose plastic containers. Am J Health Syst Pharmacy.

[24] Coyle DE, Denson DD (1986). Simultaneous measurement of bupivacaine, etidocaine, lidocaine, meperidine, mepivacaine, and methadone. Ther Drug Monit.

[25] European Medicines Agency. ICH Topic Q2 (R1). Validation of Analytical Procedures: Text and Methodology. 2006.https://www.ema.europa.eu/en/documents/scientific-guideline/ich-q-2-r1-validation-analytical-procedures-text-methodology-step-5_en.pdf (Accessed 2 May 2020).

[26] Almeida AM, Castel-Branco MM, Falcão AC (2002). Linear regression for calibration lines revisited: weighting schemes for bioanalytical methods. J Chromatogr B Analyt Technol Biomed Life Sci.

[27] Eurachem, Guidance Document No. WGD 2, Accreditation for Chemical Laboratories: Guidance on the Interpretation of the EN45000 Series of Standards and ISO/IEC, Guide 25, 1993. https://www.eurachem.org/images/stories/Guides/pdf/Eurachem_CITAC_QAC_2016_EN.pdf

[28] European Medicines Agency. Guideline on Stability Testing: Stability Testing of Existing Active Substances and Related Finished Products. 2007.https://www.ema.europa.eu/en/documents/scientific-guideline/guideline-stability-testing-stability-testing-existing-active-substances-related-finished-products_en.pdf (Accessed 2 May 2020).

[29] European Medicines Agency (EMA), Committee for Medicinal Products for Human Use. Guideline on bioanalytical method validation. 2011.https://www.ema.europa.eu/en/documents/scientific-guideline/guideline-bioanalytical-method-validation_en.pdf (Accessed 8 Mar 2020).

